# The Use of Glycerine as a Solvent in Hypodermic Injections

**Published:** 1872-06

**Authors:** 


### The Use of Glycerine as a Solvent in Hypodermic Injections.

Dr. M. Rosenthal calls attention in the *Wiener Medizinische Presse*, January 7, 1872, to the power which glycerine possesses to dissolve various of the substances which are ordinarily used in hypodermic medication. Its solvent powers are greater than those of water, and are very much increased by heat. Thus, a fluidrachm of glycerine, when heated, will readily dissolve twenty grains of the sulphate of quinia, from ten to twelve grains of the acetate or muriate of morphia, and ten grains of the extract of opium. Morphia may be added to a solution of quinia in glycerine, without causing a precipitate. It will also dissolve from half a drachm to one drachm of the iodide or bromide of potassium, and four grains of corrosive sublimate. These substances are not precipitated as the liquid cools; on the contrary, the solution will remain clear and fit for use during at least a year.—*Medical Times.*

